# Mechanisms of Peritoneal Fibrosis: Focus on Immune Cells–Peritoneal Stroma Interactions

**DOI:** 10.3389/fimmu.2021.607204

**Published:** 2021-03-29

**Authors:** Michela Terri, Flavia Trionfetti, Claudia Montaldo, Marco Cordani, Marco Tripodi, Manuel Lopez-Cabrera, Raffaele Strippoli

**Affiliations:** ^1^Department of Molecular Medicine, Sapienza University of Rome, Rome, Italy; ^2^National Institute for Infectious Diseases L. Spallanzani, Istituto di Ricovero e Cura a Carattere Scientifico (IRCCS), Rome, Italy; ^3^instituto Madrileño de Estudios Avanzados en Nanociencia (IMDEA) Nanociencia, Madrid, Spain; ^4^Istituto Pasteur Italia-Fondazione Cenci Bolognetti, Sapienza University of Rome, Rome, Italy; ^5^Programa de Homeostasis de Tejidos y Organos, Centro de Biología Molecular “Severo Ochoa”-Consejo Superior de Investigaciones Científicas (CSIC), Madrid, Spain

**Keywords:** peritoneal fibrosis, mesothelial cells, peritonitis, innate immunity, T cell subpopulations, pro-inflammatory cytokines

## Abstract

Peritoneal fibrosis is characterized by abnormal production of extracellular matrix proteins leading to progressive thickening of the submesothelial compact zone of the peritoneal membrane. This process may be caused by a number of insults including pathological conditions linked to clinical practice, such as peritoneal dialysis, abdominal surgery, hemoperitoneum, and infectious peritonitis. All these events may cause acute/chronic inflammation and injury to the peritoneal membrane, which undergoes progressive fibrosis, angiogenesis, and vasculopathy. Among the cellular processes implicated in these peritoneal alterations is the generation of myofibroblasts from mesothelial cells and other cellular sources that are central in the induction of fibrosis and in the subsequent functional deterioration of the peritoneal membrane. Myofibroblast generation and activity is actually integrated in a complex network of extracellular signals generated by the various cellular types, including leukocytes, stably residing or recirculating along the peritoneal membrane. Here, the main extracellular factors and the cellular players are described with emphasis on the cross-talk between immune system and cells of the peritoneal stroma. The understanding of cellular and molecular mechanisms underlying fibrosis of the peritoneal membrane has both a basic and a translational relevance, since it may be useful for setup of therapies aimed at counteracting the deterioration as well as restoring the homeostasis of the peritoneal membrane.

## Introduction

**Peritoneum** is a serosal membrane forming the lining of the abdominal cavity. Peritoneum is a first line of defense against microorganisms and tumor cells. Moreover, peritoneum constitutes a slippery non-adhesive surface allowing frictionless movements of the viscera in the abdominal cavity. Peritoneum is composed of a continuous monolayer of cells of mesodermal origin, the mesothelial cells (MCs). MCs cover a submesothelial region made of a thin layer of connective tissue composed mainly of bundles of collagen fibers with few fibroblasts, macrophages (MØs), mast cells, and hematic and lymphatic vessels (1, 2).

**Peritoneal fibrosis** is the end point of a progressive alteration of the peritoneal membrane due to a wide array of inflammatory and infectious events, many of which are directly related to clinical practices (3). A main cause of peritoneal fibrosis is, in fact, **peritoneal dialysis** (PD). PD is a form of renal replacement alternative to the hemodialysis, where peritoneal membrane is used as a dialysis membrane in therapeutic procedures for the treatment of end-stage renal disease. Currently, peritoneal dialysis (PD) accounts for around 10% of all forms of renal replacement therapy worldwide (4). During PD practice, signs of fibrosis are found in 50 to 80% of patients within one or two years of PD (3, 5).

Peritoneal fibrosis represents an important cause of PD discontinuation, together with peritonitis and death due to cardiovascular complications. PD is also a risk factor for the onset of **encapsulating peritoneal sclerosis** (**EPS**), the most serious complication of PD, with potentially fatal manifestation (6). EPS is a syndrome characterized by loss of ultrafiltration function, anorexia, weight loss, diarrhea, intestinal obstruction, inflammation, peritoneal thickening, fibrin deposition, sclerosis, calcification and encapsulation (7). However, peritoneum during PD practice often presents only limited complications and many patients develop a **simple peritoneal sclerosis** (**SPS**), characterized by thickening of the peritoneum, calcification, presence of inflammatory elements, angiogenesis and dilatation of blood and lymphatic vessels in the absence of systemic disease, and whose alterations are at least in part reversible after discontinuation of PD.

Besides fibrosis during PD practice, peritoneum is directly implicated in the genesis of post-surgical intra-abdominal adhesions **(peritoneal adhesions, PAs)**, which are fibrous bands tethering organs to one another or to the parietal peritoneal wall, leading to a significant cause of post-surgical morbidity and posing a major public health challenge (8). Their primary sequelae include bowel obstruction, female infertility, ectopic gestation, chronic abdominal and pelvic pain, poor quality of life, and death. It is estimated that ~93% of patients undergoing abdominal surgery develop adhesions and about 20% require re-hospitalization for adhesion-related complications (9, 10).

Finally, the insurgence of peritoneal fibrosis has a clinical relevance also for **peritoneal metastases**. In this context, metastatic tumors (generally ovary or colon cancers) instruct a fibrotic response in the peritoneal membrane, generating areas where tumor spreading and dissemination are facilitated (11–13). Although fibrosis related to peritoneal tumors is the object of increasing interest, due to its intrinsic specificities, this review article will not deal with this topic.

The induction of peritoneal fibrosis is a complex pathological event where peritoneal cells sense the pro-fibrotic stimuli and secrete extracellular mediators leading to the recruitment of circulating leukocytes playing a role in induction and amplification of the inflammatory response. The generation of myofibroblasts, cells of heterogenous origin with the ability of producing and remodeling the extracellular matrix proteins (ECM) is central for fibrosis onset. At the same time, the nature of the stimuli imparts signals promoting the resolution of the inflammatory state, with phagocytosis of dead cells and removal of debris. In this context, an implication of adaptive immunity has been proven relevant in its cross talk with peritoneal stroma or innate immunity components.

Therefore, the onset of peritoneal fibrosis is the final result of a tight network of signals between stromal resident and immune recirculating leukocytes, whose understanding may lead to a better medical containment of this deleterious pathologic event.

There is now plenty of information on the role of the non-immune components of peritoneal membrane (MCs, fibroblasts, endothelium) and activities of innate and adaptive immunity have been described by relevant studies (14); the main underlying intracellular mechanisms have been reviewed elsewhere (3, 15). The aim of this review article is to create a comprehensive synthetic description of how different signals from both stromal cells and immune system components are integrated and how cellular components are mutually influenced during the induction of peritoneal fibrosis.

## Peritoneal Fibrosis: Multiple Ingredients for One Cake

**Figure 1:**
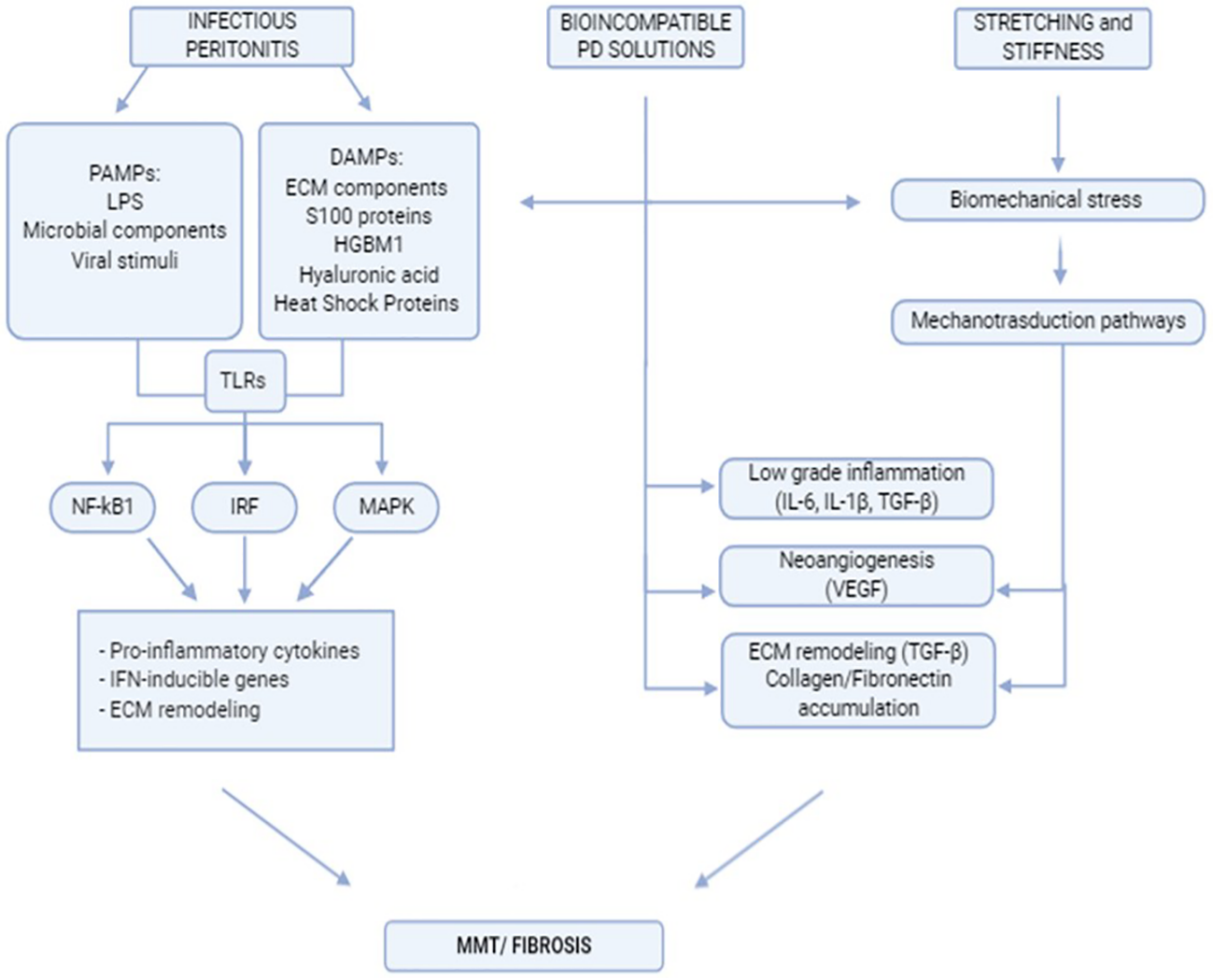
Flowchart indicating the main extracellular stimuli promoting peritoneal fibrosis and subsequent mesothelial cell response.

Peritoneal fibrosis onset is the final result of complex interactions between external stimuli, intrinsic properties of the peritoneal membrane, and subsequent activities of the local innate-adaptive immune system. A flowchart describing the stimuli discussed in this chapter and the main peritoneal stromal responses is shown in **Figure 1**.

### Infectious Peritonitis

Peritonitis is a main cause of fibrosis induction in peritoneum. Peritonitis onset is one of the most serious complication of PD: it induces angiogenesis and fibrosis and is a major cause of morbidity and mortality in PD patients (16). Repeated episodes of peritonitis are often a cause of discontinuation of PD and may precede induction of EPS (17).

Many microorganisms may infect peritoneum; the peritoneal membrane is contiguous to the intestine which harbor bacteria than can leak towards the peritoneal cavity. Moreover, medical actions such as catheter positioning and maintenance, practice of peritoneal dialysis and abdominal surgery may favor the entry of microorganisms in the peritoneum space.

The majority of peritonitis episodes in PD can be ascribed to Gram-positive bacteria of the skin and, to a minor degree, to Gram-negative bacteria presumably originating from the enteric flora (18).

Compared to bacteria, there are limited reports on the role of viruses. The suspect of virus infection occurs when cultures from peritonitis appear negative, an event occurring around 20% of the cases: however, virus infection is not diagnosed by standard tests (19).

Similarly to pericardium, coxackievirus B1 infection has been reported in peritoneum and it is characterized by the presence of monocytosis in PD effluent (20). Also, less studied are peritonitides caused by fungal infections. They constitute a serious complication of PD and account for between 1 and 15% of all PD-associated peritonitis episodes. The majority of these FP episodes are caused by Candida species such as *Candida albicans* (21, 22).

#### Mechanism of Inflammatory Response: Exogenous TLR Ligands

It is believed that the damage to the peritoneal membrane by infectious agents is mediated mainly by innate pattern recognition receptors (PRRs) on peritoneum, which include Toll-like receptors (TLRs), RIG-I-like receptors, NOD-like receptors, and C-type lectin receptors. The intracellular signaling cascades triggered by these PRRs lead to transcriptional expression of inflammatory mediators that coordinate the elimination of pathogens and infected cells (23).

Pathogens are recognized by PRRs through the interaction with molecules conserved among microbial species, which are called pathogen-associated molecular patterns (PAMPs). Besides PAMPs, PRRs also recognize endogenous molecules released from damaged cells, termed damage-associated molecular patterns (DAMPs) (24).

Among PRRs, TLRs play a critical role in innate immune responses by specifically recognizing molecular patterns from a wide range of microorganisms, including bacteria, fungi and viruses. TLRs are responsible for sensing invading pathogens outside of the cell and in intracellular endosomes and lysosomes (23). 10 different TLRs in humans and 12 in mice have been so far identified. Each of them recognizes different molecular patterns of microorganisms and self-components.

Human MCs respond to bacterial ligands through a specific subset of TLRs (*i.e.* TLR1, TLR2, TLR3, TLR5 and TLR6).

Gram positive bacteria are recognized by TLR2 and TLR5 (25), both singularly and cross-talking to better counteract microbial infections (26).

TLR2 recognizes an array of microbial molecules in part by hetero-dimerization with other TLRs (*e.g.* TLR1 and TLR6) or unrelated receptors (*e.g.* Dectin-1, CD36 and CD14). TLR activation triggers nuclear factor-kappa B (NF-*κ*B), interferon regulatory factor (IRF) and mitogen-activated protein kinase (MAPK) signaling leading to altered gene expression, including pro-inflammatory cytokine and IFN-inducible genes (27).

TLR5 recognizes flagellin, a flagellum component in many motile bacteria (28). TLR5 expression on MCs may therefore be a critical signal of flagellated bacteria’s invasion into the peritoneal cavity. Translocation of intestinal bacteria is a potential cause of infection in PD patients, along with access through the intraperitoneal catheter, and many flagellated bacteria are Gram-negative species, with a poor outcome in PD associated peritonitis (25).

Gram-negative bacteria induce responses through TLR4, initially identified as responsible for the recognition of lipopolysaccharide (LPS). Differently from murine MCs, human MCs do not directly respond to TLR4. However, TLR4 is present in MØ stably residing in the peritoneal membrane and their response may contribute to inflammation leading to fibrosis.

Recent studies have shown that the modulation of TLR2 and TLR4 activity through specific antibodies or soluble Toll-like receptor 2 (sTLR2), a TLR2 inhibitor, is able to cause a substantial reduction of inflammatory parameters to inhibit fibrosis development in an experimental model of *S. epidermidis* infection (29).

A set of TLRs, comprising TLR3, TLR7, TLR8, and TLR9, act in the intracellular space in order to recognize nucleic acids derived from viruses and bacteria, as well as endogenous nucleic acids in pathogenic contexts (23). These TLRs respond by activating the production of type I IFNs and pro-inflammatory cytokines. Viral stimuli are recognized by the intracellular TLR3, which is functionally expressed in MCs (30). While for several exogenous TLRs the signaling pathway depends on MyD88, known as the inductor of the early phase response in MØs, TLR3, specifically, acts thought TRIF that plays an essential role in inducing a NF-*κ*B mediated fibrosis and a late phase immune response activation (31, 32).

In human MCs, TLR3 is also involved in the regulation of the final common pathway of inflammation and fibrosis acting on matrix-remodeling proteins. In particular, TLR3 is correlated in time- and dose-dependent upregulation of MMP9 and TIMP1 (33).

#### Mechanisms of Inflammatory Response: Endogenous TLR Ligands

In addition to PAMPs, TLR mediated response can be stimulated by endogenous TLR molecules, inducing sterile inflammatory processes (34, 35). Many endogenous TLRs derive from ECM components, such as fibronectin or fibrinogen or ECM interacting proteins such as tenascin-C (36, 37).

Proteins with various functions may serve as endogenous TLRs such as cardiac myosin, S100 proteins, HGBM1 (38) (39–41). While the last protein may interact with several TLRs, the majority of these ligands are direct agonists of TLR2 and TLR4 (42, 43). Interestingly, exposure to PD fluids promotes the expression of Hsp60, Hsp70 and hyaluronic acid (HA), all TLR2 and TLR4 ligands, by leukocytes and MCs, thus driving an inflammatory response in the absence of infectious stimuli (see below) (44). Accordingly, treatment with soluble TLR2 (sTLR2) reduces pro-inflammatory and fibrotic response in mice exposed to PD fluids. These discoveries open to future clinical trials testing the clinical efficacy of these compound in patients undergoing long term PD (44).

### Bioincompatibility of PD Solutions

The partial bioincompatibility of fluids used for the practice of PD may act as pro-fibrotic stimuli causing progressive morphological changes and leading to functional alterations that may cause ultrafiltration failure, discontinuation of PD and increased risk of developing EPS.

Traditional PD solutions, in fact, are hyperosmotic, hyperglycemic and acid. These solutions contain sodium, chloride, calcium, magnesium, lactate and a high concentration of glucose. Low pH in these solutions counteracts glucose oxidation that may release in the solution toxic glucose degradation products (GDPs) during the sterilization process. Moreover, glucose and reactive carbonyl compounds can form Advanced Glycation End-products (AGEs), binding to free amino groups on proteins or lipids (45, 46). The high osmolarity and the high glucose concentration favor ultrafiltration and toxin elimination by keeping the electrolyte balance (47).

All these factors may promote a low-grade inflammatory status in the peritoneal membrane, characterized by increase of inflammatory and profibrotic cytokine production such as IL-6, IL-1β, TGF-*β*1, VEGF, acceleration in TIMP release, causing a loss of balance in ECM remodeling and an accumulation of collagen and fibronectin. The same factors have a cytotoxic effect on MCs inducing mesothelial denudation of the peritoneal membrane and a decrease in the intercellular junctional proteins levels, causing hyperpermeability (48). *In vitro* evidence has demonstrated that the so called ‘bioincompatible’ PD fluid may induce apoptosis of MCs (49).

More recently, *in vitro* and *in vivo* studies have demonstrated that high glucose peritoneal dialysis solutions (HGPDS) may cause apoptosis and autophagy of MCs. However, further efforts will be necessary for the full understanding of the role of these mechanisms in the genesis of fibrosis (50).

This variety of stimuli also promotes a process known as mesothelial to mesenchymal transition (MMT) (see below) contributing to matrix deposition, increased stiffness and fibrosis (51). These cellular and molecular alterations parallel the induction of numerous morphological changes in the peritoneal membrane (PM) including increased thickness of the submesothelial space, vascular changes with subendothelial hyalinization, luminal narrowing or obliteration, increased density of blood vessels (52).

Clinically, these changes reflect an increase in small solute transport due to neoangiogenesis that extends the peritoneal surface area (a blood vessel density related parameter) and ultrafiltration reduction due to fibrosis and thickness of the submesothelial zone (53, 54). In certain cases, the simple peritoneal sclerosis common in peritoneum of PD patients can lead to EPS (7).

In order to mitigate the side effects of traditional PD solutions, a second generation of so called ‘biocompatible’ PD fluids has been designed that can be divided in two main groups: PD solutions with neutral pH, low GDPs and PD solutions where glucose is replaced with glucose polymers (icodextrin) or amino acids (55–57). The functionality of PD solutions is debated. It has been reported that these solutions better preserve the residual renal function and diuresis with a decrease in peritonitis frequency (3, 58). *In vitro* and *in vivo* effects of traditional *versus* biocompatible PD fluids are summarized in **Table 1**.

**Table 1:** Table comparing the main characteristics of traditional *versus* biocompatible PD with emphasis on *in vitro/in vivo* mechanisms of toxicity.

PD solution	Traditional PD fluids	Biocompatible PD fluids		
PD solution type	Traditional PD solutions	Neutral pH, Low GDPs	Icodextrin based	Amino acid based
**Osmotic agent**	Glucose	Glucose	Icodextrin	Amino acids
**pH**	5.5	6.8-7.3	5.5	~6.7
**Toxic Agents**	GDPs, AGEs, ROS, acidic pH, lactate buffer	Significant reduction of toxic agents (GDPs, AGEs, ROS)	Acid pH, lactate buffer, ROS	High concentration of amino acids
**Mechanisms of cytotoxicity**	TGFβ and VEGF, acceleration of TIMP release, inflammatory cytokines (IL-6, IL-1β) production	↓ osmolality	Iron accumulation, ↑ maltose and maltotriose serum level	Protein accumulation
***in vitro* effects**	MMT induction, ECM deposition, increased stiffness, fibrosis, MC apoptosis	Improvement in cellular functions	pH-dependent apoptosis	Increase in nitrogenous waste metabolism
***in vivo* effects**	Peritonitis, vasculopathy, disruption of renal functions, anuria, infusion pain, diabetic glomerulosclerosis	Probable reduction in ultrafiltration; ↑ urine volume	Hypoglycemia, skin rush	Acidosis, uremia
**Benefits**	Ultrafiltration efficiency, Lower costs	Preservation of residual renal functions, reduction of peritonitis risk	Increased daily ultrafiltration, reduced glucose adsorption, increased glycemic control of diabetic PD patients, improvement of cardiac parameters	Improved surrogate markers of nutritional status of malnourished PD patients
**References**	45, 46, 48, 53, 54, 59	3, 55–58	3, 55, 56, 58, 60, 61	3, 55, 56, 58, 62–65

However, the effectiveness and the long-term benefits are currently being analyzed and there is not a definitive consensus on the benefits of this treatment, especially in the long term (66–69).

Icodextrin is a glucose polymer with a high molecular weight. PD solutions based on the use of icodextrin seem to increase peritoneal ultrafiltration, to reduce glucose absorption and to improve cardiac parameter (60). The use of a glucose polymer as an osmotic agent is particularly interesting as a glucose substitute in diabetic subjects. The reduced carbohydrate load also seems to provide a long-term metabolic advantage in terms of lipid control (61). However, icodextrin can interfere with blood glucose measurement by providing falsely elevated results. It can also cause hypersensitivity reactions, and it is more expensive than other PD solutions.

Solutions containing amino acids have been produced to improve the nutritional status of subjects on PD. PD causes a significant loss of protein in the dialysate, estimated to be 2–4 g of amino acids per day. Amino acid 1.1% solutions were found to be effective osmotic agents (62). In some studies, they have improved the nutritional status of malnourished PD patients (63). Common side effects include worsening of acidosis and an increase in blood urea linked to the increase in nitrogenous waste metabolism.

Glucose has been partially replaced by two osmometabolic agents, xylitol and L-carnitine. Treatment with this new formulation resulted a higher cell viability, better preservation of the integrity of the mesothelial layer, and reduced release of pro-inflammatory cytokines, as reported in a recent *in vitro* study (70).

Another field of investigation is the search of immunomodulators that may be added to mitigate the cellular effect of prolonged PD treatment. A recent discovery is the immunomodulatory effect of alanyl-glutamine (AlaGln) supplementation in PD solutions. This treatment seems to ameliorate peritoneal inflammation status and to improve healthy peritoneum biomarkers as well as tight junction organization and functionality (64, 65).

### PM Damage by Biomechanical Cues: Stiffness and Stretching

Besides extracellular biochemical mediators, a vast body of evidence has demonstrated a role for biomechanical forces in mediating cell physiopathological responses.

Changes in biomechanical features of the extracellular matrix (ECM), such as ECM stiffness, can modify cell state and are major promoters of a fibrotic response (71). Beyond ECM stiffness, the sensing of mechanical stretching is characteristic of organs and tissues exposed to continuous variations of dynamic cues, such as respiratory and abdominal movements or the cyclic blood circulation pulse wave. In cells with epithelial features, the effects of cellular stretching have been analyzed especially on tissues composed of monocellular layers, such as lung epithelial cells and endothelium (72, 73). Biomechanical forces affect signal transduction (mechanotransduction) with a consequent impact on cellular behavior (74).

During exposure to PD fluid, the PM experiences continuous biomechanical cues. PD practice requires the injection of large PD solution volume (2 l). This causes mechanical stress by swelling the abdominal cavity, involving mechanical stretching of MCs. Other mechanical perturbations may arise from the trauma of the peritoneal membrane after abdominal laparotomies (75).

MCs upon exposure to cellular stretch *in vitro* increase the expression of VEGF and of TGF-*β*1 (76). It has been recently demonstrated that exposure of MCs to linear cyclic stretch *in vitro* leads to several cellular modifications corresponding to bona fide MMT induction. The experimental data are summarized in a model where a cross-talk between biomechanical and biochemical signals result in the induction of MMT (77).

Biomechanical forces are also involved in the formation of PAs, with a key contribution by MCs. It is believed that in *in vivo* conditions besides the mechanical tension, also hypoxia and activation of coagulation contribute to the formation of the fibrotic response leading to PAs formation (78).

## Cellular Players of Peritoneal Fibrosis

### Stromal Components of the PM: MCs, Endothelial Cells and Stromal Fibroblasts as a Source of Myofibroblasts

Peritoneal MCs constitute a monolayer of cells with an epithelial-like cobblestone shape covering in a continuum the peritoneal cavity. MCs originate from mesoderm during the gastrulation, and their differentiation is controlled by the transcription factor WT1, which is commonly used for lineage tracing experiments (79, 80). Despite their mesodermal origin, MCs show a cobblestone morphology and actually coexpress in basal conditions epithelial and mesenchymal markers (51, 59, 81).

MCs express tight and adherent junction related molecules such as ZO-1, occludin, claudins and E-cadherin, which is expressed both in plasma membrane and in cytoplasm (82). Moreover, these cells express epithelial intermediate filament proteins such as cytokeratins (8–18) that play an important role in maintaining cellular structural integrity. At the same time, MCs constitutively express also mesenchymal intermediate filaments such as vimentin and desmin (51).

The coexistence of both epithelial and mesenchymal markers may be linked to the characteristic plasticity and to the ability of these cells to acquire mesenchymal-like features in response to a variety of pro-inflammatory/profibrotic stimuli. Almost all the pro-inflammatory factors described in the previous sections may promote, although with different intensity, induction of MMT in MCs. This dedifferentiation process culminates with the acquisition of morphological and functional features making these cells indistinguishable from myofibroblasts of other origin (see below) (**Table 2**).

**Table 2:** Epithelial-like and mesenchymal markers of MCs. The main extracellular regulators of MC plasticity, molecular markers and signaling pathways implicated are shown.

Epithelial-like MCs					
Features and Properties	Extracellular Mediators		Markers	Trascription Factors and Signaling Pathways	
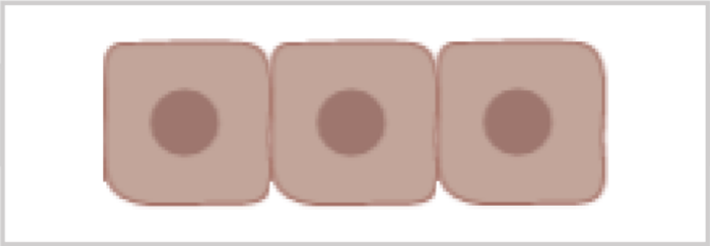	BMP7		E-Cadherin	WT1	
	BMP4		Claudins	SMAD1-5-8	
	IGFBP4		Occludins	p38 MAPK	
Cobblestone-like shape	HGF		ZO-1		
Apical-basal polarity			Desmoplakin		
Monolayer organization			Cytokeratins		
Tight junctions			Calretinin		
Adherens junctions			Vimentin		
Glycocalyx production			VEGFR2		
Immunomodulatory activity			CA125		
			Caveolin-1		
			Hyaluronan		
References	(83, 84)		(51, 77, 82, 85–90)	(79, 80, 82, 91)	
**Mesenchymal-like MCs (MMT)**					
**Features and Properties**	**Extracellular Mediators**	**Markers**		**Trascription Factors and Signaling Pathways**	
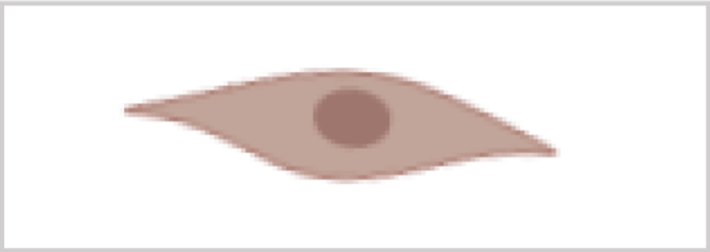	TGFβ1	N-Cadherin		Snail, Twist	
	IL-1β	Desmin		SMAD 2-3	
	FGF-2	Vimentin		GSK-3β	
Spindle-like shape	EGF	Fibronectin		Wnt/β-Catenin	
Front-back polarity	AngII	Collagen I/III		TAK1/NF-κB	
Junctions dissociation	AGEs	α-SMA		ILK	
Cadherin switch	PDGF	FSP-1		PI3-K	
Cytoskeleton reorganization	HIF-1α	MMPs (2-9)		ERK 1/2 MAPK	
ECM deposition		PAI-1		JNK 1/2 MAPK	
Basement membrane degradation		Podoplanin			
Migratory and invasive activity		CTGF			
Proinflammatory activity					
References	(92–98)	(75, 77, 78, 82, 99–101)		(59, 102–106)	

The secretion of TGF-*β* by MCs or by other cells such as MØs is central for a full induction of the MMT program. Once transdifferentiated, MCs may invade the submesothelial stroma where they proliferate and produce cytokines and ECM proteins directly promoting peritoneal fibrosis.

The profibrotic activity of TGF-*β*1 is counteracted by members of the BMP family, such as IGFBP4, BMP4 and BMP7, secreted by the same MCs (83, 84).

Interestingly, transdifferentiated MCs tend to acquire a new stability. This behaviour is different from that of cells with a stronger epithelial identity that rapidly recover epithelial features once the transdifferentiating stimulus has been removed (107). The maintenance of mesenchymal features in MCs has been linked to epigenetic changes, and epigenetic modulation may both influence mesothelial differentiation and promote the recovery of a “epithelial-like” phenotype from *in vivo* transdifferentiated cells (99, 108).

High throughput experiments have demonstrated that induction of MMT from different stimuli induces the acquisition of common dedifferentiation features characterized by the expression of signatures of profibrotic and pro-inflammatory cytokines such as TGF*β*, VEGF, and IL-6 (29, 77, 83, 109). In fact, activated MCs are major producers of TGF-*β*1, VEGF and IL-6, whose concentrations are elevated especially during peritonitis and have been associated with ultrafiltration decline and protein loss (18, 110). The secretion of these cytokines impacts fibrosis, angiogenesis and the inflammatory response.

The ability of MCs to generate myofibroblasts has been a highly debated topic in the previous years. Lineage tracing experiments performed to demonstrate the mesothelial origin of peritoneal myofibroblasts have given contrasting results, with the more recent studies suggesting the existence of a population of MCs origin invading the submesothelial space (83, 111, 112).

Moreover, the coexpression of *bona fide* MMT markers such as αSMA and fsp1 absent in epithelial-like MCs with mesothelial/epithelial markers has been demonstrated *in vivo* both in peritoneum after PD and in peritoneal adhesion (75, 77, 78, 82).

In the peritoneum of mice exposed to PD fluid, the relative contribution of the myofibroblasts-generating cellular populations, including resident dermal fibroblasts, endothelial cells, bone marrow derived cells and MCs, has been quantified (113). As in other organs, endothelial cells contribute to peritoneal fibrosis through a process of endothelial to mesenchymal transition (EndMT) (82, 114). Also, bone marrow derived progenitors, such as mesenchymal stem cells and fibrocytes may generate peritoneal myofibroblasts (115).

MCs may in different ways influence the fibrotic process. Besides being a main source of TGF-*β*1, activated MCs produce abundant amounts of fibronectin and collagens, may rearrange the ECM through the expression of contractile proteins (*α*SMA) and produce various metalloproteinases (MMPs), such as MMP2, MMP9 and MMP14 as well as MMP inhibitors such as TIMP1 and PAI1 (77, 99, 100). Besides directly impacting the fibrotic process, the production of inflammatory cytokines and chemokines stimulates other stromal cells and components of innate and adaptive immunity (see below). Thus, MCs are candidates for cellular interventions aimed at restoring the continuity of the monolayer and to warrant the peritoneal function.

## Leukocyte Subpopulations Implicated in the Fibrotic Response

### Peritoneum as a Lymphatic Organ: The Role of FALCs

Due to its unique localization in the abdominal cavity and its huge extension, peritoneum is a favorite site for encountering with antigens and for the generation of the subsequent immune response. Recirculating leukocytes patrol the peritoneal cavity in uninflamed peritoneum in addition to stably resident leukocyte populations constituted mainly by macrophages and mast cells.

Besides conventional lymph nodes, peritoneum hosts unique anatomic structures called milky spots or **fat-associated lymphoid clusters** (**FALC**s), which are clusters of leukocytes localized especially in omentum and endowed with the ability to collect fluids, particulates, and cells from the peritoneal cavity. Their frequency and size increase in the omentum of patients undergoing PD (116, 117). FALCs are mainly composed of MØs, MCs and B1 cells. B1 cells consist in a subset of B cells that can be distinguished from conventional B (B2) cells by expression of distinct cell-surface markers and antigen receptors that can bind common bacterial epitopes. B1 cells have the potential to produce natural antibodies that provide a first protection to bacterial infections (118). Intestinal leakage or the intraperitoneal delivery of microorganisms lead to rapid activation of B1 cells and promote T cell-independent antibody responses (119).

**Figure 2:**
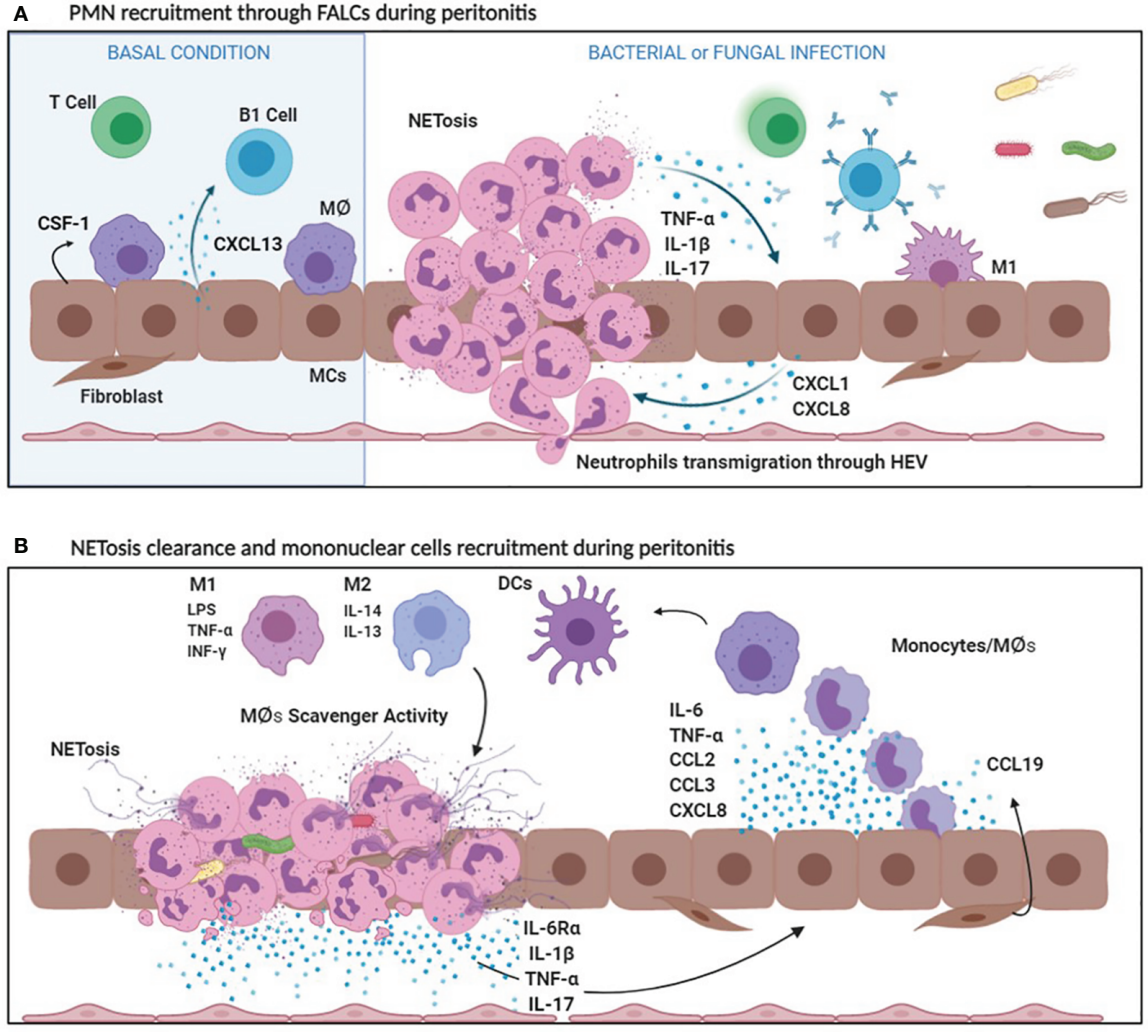
**(A)** PMN recruitment through FALCs during peritonitis. In basal conditions, MCs secrete CXCL13, which attracts B1 cells in FALCs and CSF1, a specific MØ growth factor. Bacterial and fungal infections stimulate the production of CXCL1 and CXCL8, by MCs. Bacterial products, CXCL1 and CXCL8 promote the recruitment of a first wave of PMNs entering the peritoneal cavity through FALCs. PMNs cause an initial inflammatory response secreting inflammatory cytokines (IL-1ß, TNF-α). Afterwards, NETosis helps in sequestering microorganisms in FALCs. **(B)** NETosis clearance and mononuclear cell recruitment during peritonitis. Bacterial products, as well as IL-1ß, stimulate the production of IL-6, TNF-α, CCL2, CCL3, and CXCL8 by MCs. IL-6Ra shedding by PMNs promotes a peripheral IL-6 response (transignaling). Cytokines and chemokines released during the inflammatory process favor mononuclear recruitment and differentiation. Mononuclear phagocytes differentiate in Macrophages (MØs) and dendritic cells (DCs). Among MØs, M1 subtype is endowed with pro-inflammatory and cytotoxic properties, whereas M2 MØs have an anti-inflammatory activity. Moreover, M2 MØs play a key role in the clearance of neutrophils debris due to scavenger activity.

The chemokine CXCL13, of mesothelial origin, controls the localization of B1 cells into FALCs (120). Another chemokine, CCL19, is produced by other structural components of FALCs called FALCs fibroblast reticular cells (FALCs FRCs) and it is relevant for monocyte recruitment during inflammation. The cross-talk between CCL19 producing FALCs FRCs and inflammatory monocytes promote T cell dependent-B cell immune responses (121) (**Figure 2A**). Thus, FALCs play a main role both in the regulation of PMN and mononuclear cell recruitment in the first phase of inflammation, as well as in the subsequent induction of the adaptive immunity.

### Leukocyte Recruitment During Peritonitis: From Neutrophils to Mononuclear Cells

Infectious peritonitis offers a favorite experimental model to study the interactions between immune system and the peritoneal stroma (122). Infection with Gram positive bacteria such as Staphylococcal spp. or with cell-free components such as LPS or zymosan, mimicking Gram negative or fungal infection, respectively, promotes a first wave of polymorphonuclear neutrophils recruited by chemoattractants of bacterial origin and by chemokines such as CXCL1 and CXCL8 produced mainly by MCs and omental fibroblasts. Neutrophils can use high endothelial venules present in FALCs to enter the peritoneal cavity under the guidance of CXCL1 (120).

Neutrophil influx causes an initial inflammatory response due to the accumulation of neutrophil-secreted proteases and reactive oxygen species. Once they entered the peritoneum, neutrophils undergo **NETosis**, which consists in the release of necrotic cell DNA forming a net of aggregated neutrophils able to trap and sequester microorganisms in FALCs, thus limiting their spreading (123). Interestingly, HMGB1 produced during the inflammatory response promotes NETosis and trap formation through interaction with TLR4 (124) (**Figure 2B**).

The production of CXCL1 and CXCL8 by the peritoneal stroma is enhanced by inflammatory cytokines such as IL-1β and to a lesser extent, TNFα (125). MCs stimulated by LPS or IL-1β also produce a number of cytokines and chemokines including IL-6, TNFα, CCL2, CCL3, that favor mononuclear cell recruitment and activation (126). The first wave of neutrophils is then replaced by a mononuclear infiltrate.

Neutrophils take part in this process secreting a shed form of IL-6 receptor, IL-6R*α*. Through a mechanism called **transignaling**, the local increase of IL-6R*α* promotes an IL-6-mediated neutrophil clearance subsequent to mononuclear cell recruitment (127, 128). Apoptotic neutrophils are phagocytosed by MØs and to a lesser extent by the same MCs (129). Necrotic neutrophils and NETs promote the infiltration of mature MØs recruited also by locally produced chemokines such as CXCL8 and CCL2 (130).

Neutrophil influx and clearance are also regulated by two other cytokines, IL-17 and IFN-γ. IL-17 is secreted mainly by various leukocyte subpopulations, including neutrophils, Th17 and *γδ* T lymphocytes and its expression correlates with the duration of the PD treatment and with the extent of peritoneal inflammation and fibrosis (131, 132). IFN-*γ* production by Th1 lymphocytes and NK cells (see below) contributes with IL-6 in favoring an initial neutrophil recruitment and subsequent clearance (133).

IL-17 promotes CXCL1 production by MCs through expression and activation of the transcription factor Sp1, whereas IFN-*γ* through STAT1 activation limits Sp1 induced CXCL1 production. Breaking of this homeostatic cross regulation may lead to excessive or ineffective recruitment of neutrophils during peritonitis with subsequent damages in the PM (131).

### Inflammation, Scavenging and Antigen Presentation: Monocytes/MØs and Dendritic Cells

Tissue mononuclear phagocytes, comprised mainly of MØs and dendritic cells (DCs), are key tissue-resident components of the peritoneal immune system. Their roles include induction of the inflammatory response, pathogen clearance, tissue repair, and antigen presentation.

MØs are the major resident immune population in the PM. At the same time, monocytes/MØs are the predominant cell types found in dialysis effluent (134, 135).

Resident MØs form the first line of defense against peritoneal infection in peritonitis. Once the inflammatory response is initiated, monocytes follow a first wave of leukocytes composed mainly by PMN neutrophils.

MØs are generally classified into two functional subtypes. Classical active MØs, also called M1 MØs (representative markers: iNOS and CD80) are defined by their pro-inflammatory and cytotoxic properties, while M2 MØs (representative markers: CD163, CD206 and Arg1) are characterized by anti-inflammatory and scavenging properties (136). However, MØ M1 and M2 subtypes should be considered as the extreme points of a continuum of different cellular populations acting in in different physio-pathological contexts (137, 138).

M1 polarization typically involves IFN-*γ* with a TLR agonist, such as LPS. M1 MØs, through production of IL-1β and TNF-α, are capable of amplifying the first phase of the inflammatory process and of recruiting other leukocytes into the peritoneum via the creation of a gradient of chemotactic cytokines, such as CXCL8, CCL2 and CCL5. This process is also facilitated by a cytokine driven up-regulation of adhesion molecule expression (ICAM-1 and VCAM-1) on the surface of MCs. At the same time, the generation of M2 MØs, which is sustained by IL-4 and IL-13, plays a role in the resolution of inflammation through the production of soluble anti-inflammatory mediators, and the clearance of debris such as apoptotic or necrotic products, due to their scavenger activity (137).

**Figure 3:**
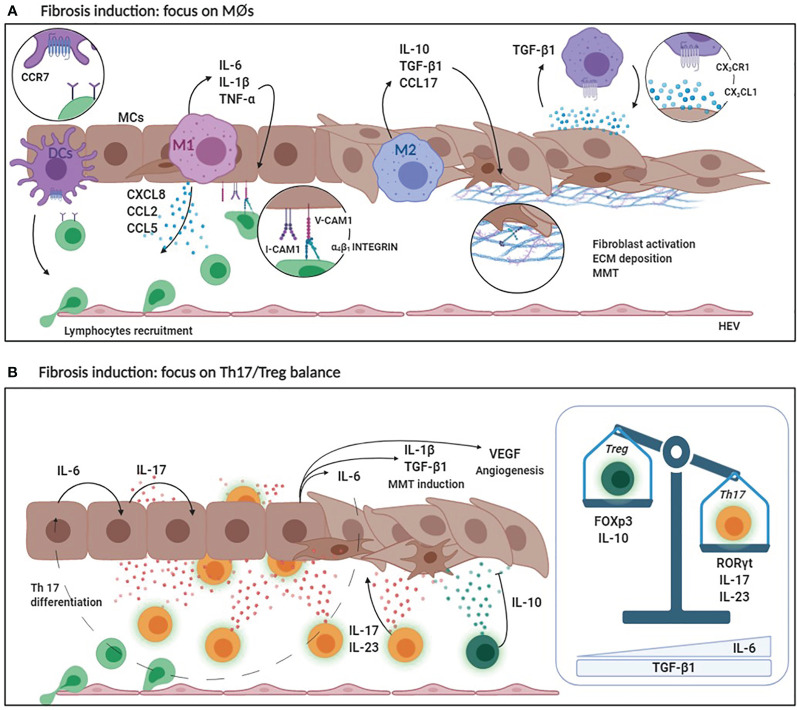
**(A)** Fibrosis induction: focus on MØs. DCs secrete high levels of CCR7 favoring lymphocyte recruitment. Pro-inflammatory M1 MØs secrete CCL2, CCL3 and CXCL8 that are chemoattractant for lymphocytes and monocytes. At the same time, M1 MØs produce inflammatory cytokines such as IL-6, IL-1ß and TNF-α that enhance the expression of adhesion molecules (V-CAM1/I-CAM1) by MCs promoting leukocyte adhesion. M2 MØs produce anti-inflammatory cytokines (IL-10) and lymphocyte chemoattracting chemokines (CCL17). Predominance of M2 MØ response leads to an increased TGF-ß1 secretion that induces MMT of MCs with up-regulation of ECM protein production. Moreover, MCs secreting CX3CL1 recruit MØs expressing CX3CR1. Receptor/ligand interaction determines a positive loop that promote, in turn, CX3CL1 and TGF-ß1 expression. **(B)** Fibrosis induction: focus on Th17/Treg balance. The production of IL-6 by MØs and MCs during the inflammatory process promotes IL-17 production by the peritoneal stroma which, in combination with IL-23, promotes the differentiation of Th17 lymphocytes. IL-17 promotes IL-1ß, TGF-ß1, VEGF and IL-6 production causing MMT induction and neoangiogenesis. Th17 abundance affects the activity of regulatory T lymphocytes. Treg lineage has an anti-inflammatory activity (due to IL-10 production) and protects the peritoneal membrane by mediating tolerance mechanisms. High levels of IL-6 and TGF-*ß*1 determine the predominance of Th17 over Treg with consequent peritoneal damage and fibrosis.

In the model of peritonitis induced by zymosan, mimicking fungal infection, an infiltration of both M1 and M2 MØs occurs. In this context, MØs are involved in the clearance of debris resulting from neutrophil apoptosis. Both M1 and M2 MØs recognize and endocytose dead cellular debris through apoptosis inhibitor of macrophage (AIM, also called CD5L), a member of the scavenger receptor cysteine-rich superfamily (22) (**Figure 3A**).

Experimental evidence analysing samples from PD patients demonstrates that the majority of peritoneal MØs phenotypically and functionally resemble *in vitro* polarized M2 MØs (139, 140).

In this context, a dysregulated M2 MØs response may promote the development of fibrosis and the decrease of functionality of the PM through the production of a number of extracellular factors.

The production of TGF-*β*1 by M2 MØs mediates the induction of MMT in MCs and the proliferation and activation of submesothelial fibroblasts, a process leading to ECM production, rearrangement, angiogenesis, and fibrosis. Moreover, MMP9 and CCL18 secretion is increased in both PD effluents and PM biopsies of PD patients (139). MMP9 plays a role in the activation of latent TGF-*β*1, whereas CCL18 levels have been associated with poor ultrafiltration capability and with development of EPS (139). M2 MØs may favor a fibrotic response also producing CCL17, which promotes migration, proliferation and collagen production by submesothelial fibroblasts (141).

However, besides M2, some evidence points towards an active role of the M1 subtype in the genesis of peritoneal fibrosis. The inhibition of the protein kinase C beta pathway promotes peritoneal damage and fibrosis *via* M1 MØs polarization in a murine model of PD (142). Also, *in vivo* approaches of MØ chemical deletion followed by reperfusion pointed towards an active role of the M1 subpopulation in the genesis of fibrosis, as suggested also by an *in vitro* study (143, 144).

The presence of MØs in the PM is tightly dependent on a cross talk with MCs. In basal conditions, MØs require and interact with MCs present in FALCs and secreting colony stimulating factor 1 (CSF1), a specific MØ growth factor (145). In inflammatory conditions and during PD, fractalkine (CX3CL1), a chemokine secreted by MCs, recruits and activates MØs expressing CX3CR1 in the peritoneal wall, promoting the fibrotic process. A positive feedback loop is induced where direct interaction with CX3CR1-expressing MØs promotes expression of CX3CL1 and TGF-*β*1 by MCs. In turn, TGF-*β*1 upregulates CX3CR1 expression in MØs (146).

Besides MØs endowed with pro-inflammatory or scavenger abilities, migrating mononuclear cells may differentiate in DC subsets, characterized by CD1c positivity and different profiles with respect to CD14 positive cells. In particular, CD1c positive cells have upregulated costimulatory molecules, CD80 and CD86, important for antigen presentation and T-cell activation, and CCR7, favoring migration to secondary lymphoid organs such as local lymph nodes where antigen presentation may occur (134). Interestingly, severe and recurrent episodes of peritonitis were associated with significantly higher numbers of peritoneal neutrophils, MØs as well as higher ratio of MØs to DCs than the successfully treated ones (134). The functional role of MØs has been analyzed with pharmacological but not with genetic or immunological approaches. Chemical depletion of MØs using clodronate attenuated peritoneal thickening and suppressed TGF-*β*1, VEGF expression and MMT induction in a model of peritoneal fibrosis induced by chlorhexidine gluconate in rats (147). Accordingly, depletion of MØs limited fibrosis in a mouse model of PD fluid exposure (143).

### Mastocytes

Mastocytes or mast cells are predominantly localized at sites that have direct contact with the external environment, such as the skin, airways, and intestine, where they function as sentinel cells in host defense and as main inducer of type I hypersensitivity and of the allergic response (148).

While mast cells have been implicated in fibrogenesis, angiogenesis, and the immune response against bacteria in various organs such as kidney and in lung, only a few studies have dealt on the role of mastocytes in peritoneal fibrosis (149, 150).

Mechanistically, mast cells secrete various mediators of inflammation such as histamine, platelet-activating factor, prostaglandins, thromboxane, leukotriene, chymase. Moreover, the secretion of cytokines such as TGF-β1 and IL-17 directly contributes to peritoneal fibrosis (149, 151, 152)

The number of mast cells was significantly higher in the fibrotic peritoneum of rats with chronic renal failure (CRF rats). Tranilast, an anti-allergic drug with an activity of mast cell stabilizer, was demonstrated to block the progression of peritoneal fibrosis in CRF rats (153).

An interesting study performed on mast cell-deficient rats demonstrated that mast cells promoted the increase of the omental thickness and omental adhesion formation favoring leukocyte recruitment (154).

Results in human peritoneal disease are controversial. A first study showed reduced numbers of mast cells in samples from PD patients (155), whereas increased mastocytes numbers have been found in samples from different inflammatory and fibrotic peritoneal diseases, including PD and EPS (156). Thus, although evidence suggests that these cells may amplify the inflammatory response during peritoneal damage, their functional role has not so far been demonstrated.

### Natural Killer Cells

Natural killer (NK) cells are a specialized lymphocyte subpopulation that play a significant role during viral infections and in tumor immune surveillance through direct killing of virus infected or tumor cells or by production of cytokines and chemokines. NK cells recirculate throughout the peritoneal cavity and are present in the peritoneal fluid. Moreover, in uninflamed peritoneum, a resident NK cell population isolated in mice was able to secrete IFN-*γ*, GM-CSF, and TNF-α and endowed with killing ability (157).

During an acute inflammatory process such as peritonitis, NK cells produce inflammatory cytokines such as TNFα and IL-6. Moreover, through production of IFN-*γ* and TGF-*β*1 these cells may directly orchestrate the fibrotic process.

In other organs, NK cells actively contribute to the genesis of the fibrotic damage. Tubulointerstitial human CD56^bright^ NK cells correlate with loss of kidney function and with induction of fibrosis and chronic kidney disease progression, mechanistically linked to increased NK cell-mediated IFN-*γ* production (158).

Interestingly, besides its potential in the amplification of the inflammatory response, NK cells appear to have a role in the resolution of inflammation in antigen-dependent peritonitis promoting neutrophil apoptosis (159). Previous results confirm that NK cells are capable of inducing apoptosis of neutrophils (160).

Studies performed in humans are limited to adoptive transfer of activated NK cells in an autologous NK cells setting used in a frame of tumor therapy. It was demonstrated that administration of NK cells in combination with IL-2 in patients with malignancies caused peritoneal fibrosis (161). Thus, although their role is potentially relevant, no definitive information is reported about NK cells in the genesis of peritoneal fibrosis.

### T Lymphocytes: A Balance of Mutually Influencing Subpopulations

Besides the components of the innate immunity, the activity of different T lymphocyte subsets is fundamental for the regulation of the inflammatory response in the genesis of peritoneal fibrosis and it could provide molecular targets to control peritoneal damages. The relevance of the adaptive immunity in peritoneal fibrosis is demonstrated by the use of lymphocyte-deficient mice. In RAG-deficient mice, lacking mature T and B lymphocytes, treatment with zymosan induced an exaggerated inflammatory response with increased PMN infiltration (162). Accordingly, the use of another experimental system demonstrated a role of adaptive immunity in limiting PMN and MØ recruitment (163).

More generally, these approaches suggest that a network of mutual interactions occurs between peritoneal stroma, innate and adaptive immunity effectors during the genesis of peritoneal fibrosis.

The composition of peritoneal fluid lymphocytes varies with respect to blood lymphocytes. In particular, B lineage comprises only around 2% of the total fluid, and T leukocyte subpopulations are differently represented (164). Moreover, changes in the consistency of T lymphocyte subpopulations occur during peritoneal inflammation and during the practice of PD.

With respect to the balance between CD4^+^ T-helper 1(Th1) and T-helper 2 (Th2) subpopulations, it has been demonstrated that during episodes of acute peritonitis, the immune response is predominantly directed to the induction of Th1 cells (165).

On the other hand, the Th2 subset rapidly expands with the practice of PD (166). The Th1/Th2 ratio could be evaluated by measuring IFN-*γ* (Th1 subset) and IL-4 (Th2 subset) levels both in circulating and peritoneum-derived Th lymphocytes. In PD patients, the IFN-*γ*/IL-4 ratio is significantly reduced, indicating a negative effect of bioincompatible fluids towards the Th1 cell subset. Interestingly, this effect could be avoided using more biocompatible fluids containing bicarbonate-buffered and icodextrin, that may reestablish a more physiological Th1/Th2 balance and a reduced peritonitis rate (167).

A recently characterized leukocyte subpopulation, Th17 lymphocytes have been demonstrated as the main driver of peritoneal fibrosis (132). The expression and the activity of this lymphocytic subset is linked to the production of IL-17. Besides Th17, other leukocytes, including CD4+ and *γδ* T lymphocytes, neutrophils, and mast cells may secrete this cytokine during exposure to PD fluids or during peritonitis (168).

The strong stimulation of Th17 response during these pathological conditions is due to both exogenous and endogenous factors. Exogenous factors are represented by bacteria and their derivatives entering the peritoneal cavity through PD catheter or *via* intestinal translocation. These bacteria stimulate TLR’s response by the peritoneal stroma, which leads to an upregulation of IL-6 levels, promoting IL-17 production and subsequent differentiation of Th17 lymphocytes (169). Similarly to bacterial derivatives, also factors related to PD fluid such as AGEs expressed in conventional lactate-based PD solution with low pH and high GDP contents are able to stimulate the Th17 response (170) (**Figure 3B**).

IL-17 contributes to the host defense against bacteria and fungi (171). It promotes neutrophil recruitment favoring the release by MCs of chemotactic factors specifically attracting neutrophils. Moreover, the IL-17 response favors the secretion of a network of cytokines and chemokines including IL-1β, IL-6, CCL2 and TGF-*β*1. In peritoneum, IL-17 favors through different mechanisms the secretion of VEGF by MCs, promoting angiogenesis (168). Most importantly, repeated intraperitoneal administration of exogenous IL-17 led to increased expression of several fibrosis-related genes, whereas its neutralization with anti-IL-17 alleviated the extent of peritoneal fibrosis (132).

The consistence of Th17 population in the peritoneum directly affects the activity of another T cell subset, regulatory T lymphocytes (Treg) (14). Tregs are suppressors of activated T cell expansion, their activity is anti-inflammatory and favors the induction of tolerance (172). IL-6, in combination with TGF-β1, is the main cytokine involved in the helper 17/regulatory T (Th17/Treg) balance. The predominance of IL-6 favors the generation of Th17 lymphocytes, which produce inflammatory cytokines. On the other hand, TGF-*β*1 in the absence of IL-6 promotes the Treg lineage, able to maintain peripheral tolerance and produce anti-inflammatory mediators such as IL-10, which has been linked to protection of the peritoneal membrane from inflammatory damage (173).

Interestingly, the plasma membrane receptor CD69 appears to control Th17/Treg balance. The exacerbated peritoneal fibrosis observed in CD69^−/−^ mice could be alleviated by the blockade of IL-17 (174). Mechanistically, it was demonstrated that CD69 directly interacting with Jak3/STAT5 blocks Th17 differentiation (175).

Besides shaping the immune response, these changes may impact MC plasticity: it was demonstrated that IL-17 itself is able to induce EMT in bronchial cells while inducing peritoneal fibrosis *in vivo* (132, 176). In contrast, low levels of IL-6 may promote Treg differentiation, which is associated to high IL-10 expression, leading to the establishment of an anti-inflammatory state and possibly MMT reversal (14, 177–179).

In case of peritoneal dialyzed patients, the predominassnce of Th17 over Treg favors fibrosis development and PM failure instead of Treg-mediated tolerance. Currently, the modulation of the expression of the cytokines involved in Th17/Treg balance through recombinant antibodies or cytokines is an attracting field for the design of new therapies aimed at counteracting peritoneal MMT and fibrosis.

A bridge between the adaptive and the innate arm of the immune system is constituted by Mucosal-associated invariant T (MAIT) cells. These cells are different from conventional T cells, since they do not react through major histocompatibility complex (MHC) (180). Peritoneal MAITs (pMAITs) provide a marker for systemic inflammation during spontaneous bacterial peritonitis (SBP), since they are configured to respond pro-inflammatory chemotactic signals sensed by CCR5, CXCR3 and CCR6 (181). pMAITs are a source of IL-17 (102). Clinical data indicated a specific immune activation of pMAIT, driven by CD69 expression and correlated to disease severity (181).

## Conclusions

The cellular and molecular mechanisms described above witness the complexity of the physiopathologic response occurring in the inflamed peritoneum.

A study published almost 25 years ago by Topley et al. identified the relationship between MØs and MCs as a key factor in the response of peritoneum to infections, whose dysregulation was causal to ultrafiltration failure and fibrosis in PD patients (182). At that time, it was already clear that MCs under MØ-driven stimuli may produce a number of extracellular mediators including arachidonic acid derivatives, cytokines and chemokines promoting the amplification of the inflammatory response. Since then, the understanding of cellular and molecular mechanisms underlying has evolved considerably. A relevant discovery has been the characterization of different MØ subpopulations implicated in the secretion of pro-inflammatory mediators, phagocytosis, apoptotic debris removal and scavenging activity. Another major breakthrough has been the identification of the ability of MCs and other cells to undergo deep dedifferentiation processes culminating in the generation of myofibroblasts. Moreover, a relevant concept that has emerged in these years is that the relationship between MØs and MCs is not unidirectional: MCs play an active role in influencing MØ recruitment, survival and differentiation due to the synthesis of extracellular mediators acting specifically on MØs. Last, this dialogue is not limited to MØs and MCs: stromal driven signals such as IL-17, IL-6 and TGF-*β*1 shape the Th17/Treg balance, thus impacting the fibrotic response.

Although the fibrotic process has common marks in all the organs, peritoneum fibrosis has specific features due to the anatomic localization and the cellular components forming this organ. The anatomic localization favors the encounter with microorganisms through unique structures (*i.e.* the FALCs) With regard to peritoneal stroma, the characteristic plasticity of MCs, their ability to transdifferentiate and to become indistinguishable from myofibroblasts, makes the difference with respect to other organs, such as the liver, where parenchyma cells (hepatocytes) may give little direct contribution in the genesis of fibrosis.

These new discoveries related to cellular communication and cellular plasticity may have an impact in future therapeutic strategies. Future directions aimed at improving peritoneal viability and thus duration of PD therapy may be focused at further improving PD fluid biocompatibility, as well at using inhibitory monoclonal antibodies, in line with recent advances with therapy of inflammatory/profibrotic diseases. Also specific therapies aimed at supporting MC viability and regulating peritoneal immune system and immune cell/MC interactions may give a contribution. Deepening the analysis of cellular and molecular mechanisms underlying peritoneal fibrosis may shed light on our understanding of how we can preserve the long-term function of the PM as a dialysing organ, but also to treat other forms of peritoneal fibrosis such as post-surgical adhesions or tumor related-peritoneal fibrosis.

## Data Availability Statement

The original contributions presented in the study are included in the article/supplementary material. Further inquiries can be directed to the corresponding author.

## Author Contributions

MTe wrote the manuscript and composed the figures. FT wrote the manuscript and organized the tables. CM wrote the manuscript. MC wrote the manuscript and composed the figures. MTr critically reviewed the manuscript. ML critically reviewed the manuscript and provided a general scheme of interpretation. RS conceived and wrote the manuscript, and critically reviewed the manuscript. All authors contributed to the article and approved the submitted version.

## Funding

Sapienza University of Rome RG11916B6A9C42C7 to MT. IMPROVE-PD project that has received funding from the European Union’s Horizon 2020 Research and Innovation Programme under the Marie Sklodowska-Curie grant agreement number 812699 to ML-C. Spanish Ministry of Science and Innovation/Fondo Europeo de Desarrollo Regional (PID2019-110132RB-I00/AEI/10.13039/501100011033) to ML-C.

## Conflict of Interest

The authors declare that the research was conducted in the absence of any commercial or financial relationships that could be construed as a potential conflict of interest.
